# The association between a fracture risk tool and frailty: Geelong Osteoporosis Study

**DOI:** 10.1186/s12877-020-01595-8

**Published:** 2020-06-05

**Authors:** Monica C. Tembo, Kara L. Holloway-Kew, Mohammadreza Mohebbi, Sophia X. Sui, Sarah M. Hosking, Sharon L. Brennan-Olsen, Lana J. Williams, Mark A. Kotowicz, Julie A. Pasco

**Affiliations:** 1 Epi-Centre for Healthy Ageing, Deakin University, PO Box 281, Geelong, Victoria 3220 Australia; 2 Faculty of Health, Biostatistics Unit, Deakin University, Geelong, VIC Australia; 3 Centre for Medicine Use and Safety, Faculty of Pharmacy and Pharmaceutical Sciences, Monash University, Parkville, Australia; 4 Australian Institute for Musculoskeletal Science (AIMSS), The University of Melbourne, St Albans, Australia; 5 Department of Medicine-Western Campus, The University of Melbourne, St Albans, Australia; 6 School of Health and Social Development, Deakin University, Geelong, VIC Australia; 7 Barwon Health, Geelong, Australia

**Keywords:** Frailty, FRAX, Association, Older adults

## Abstract

**Background:**

Frailty is characterised by age-related declines in physical, psychological and social functioning. Features of frailty overlap with risk factors for fragility fractures. The aim of this study was to investigate the association between the fracture risk assessment tool (FRAX®) and frailty.

**Methods:**

In cross-sectional analysis, frailty status was determined for participants aged 60-90 yr at 15-year follow-up of the Geelong Osteoporosis Study, using a modified Fried frailty phenotype. Using the FRAX on-line tool, scores for hip and major osteoporotic fracture (MOF) were calculated with and without bone mineral density (BMD). Using the area under Receiver Operating Characteristic (AUROC) curves, and FRAX scores calculated at the baseline visit for these participants, we investigated the association of FRAX and frailty 15 years later.

**Results:**

Forty-seven of 303 women (15.5%) and 41 of 282 men (14.5%) were frail at the 15-year visit. There was a gradient of increasing median FRAX scores from robust to frail. For example, for women, median MOF-FRAX without BMD increased from 5.9 for the robust to 7.5 for the pre-frail and 14.0 for the frail (*p* < 0.001). In secondary analyses, an association was observed between FRAX and frailty over 15 years, with the highest AUROC for women being 0.72 for MOF-FRAX with BMD, and for men, 0.76 hip-FRAX without BMD.

**Conclusion:**

An association was observed between FRAX and frailty where frail men and women had higher FRAX-scores compared to the other groups. Preliminary data suggest that FRAX, with or without BMD, may be useful in enhancing the information on frailty. Further research using larger datasets will be required to explore this.

## Background

Frailty is a clinical syndrome characterised by age-related declines of physical, psychological and social functioning [1, 2]. Frailty increases vulnerability to adverse health outcomes, such as falls, fractures, hospitalisation and disability, associated with diminished ability to compensate for disruptions in homeostasis and minor stressors [3, 4]. Prevalence estimates for frailty vary due to the heterogeneity among the frailty assessment tools currently available. The most commonly used and widely validated tools for assessing frailty are the frailty phenotype and frailty index of deficit accumulation [5].

Frailty is associated with body compositional changes, sarcopenia and osteoporosis, having overlapping pathogenic pathways related to loss of lean muscle mass and function and skeletal deterioration [6]. Osteoporosis is characterised by low bone mineral density (BMD) and deteriorated microarchitecture [7] resulting in increased bone fragility and fracture risk. Emerging studies suggest that frailty may be an effective predictor of osteoporotic fracture [8, 9], since individuals with severe frailty have an increased likelihood of prevalent and incident fractures [7, 10]. Previous studies have reported that measures of frailty can predict the risk and occurrence of falls and fall-related fractures [3, 8, 9, 11]. There is also an overlap between the characteristics of individuals with frailty and fragility fracture [10].

With the afore mentioned overlap between frailty and fragility fractures, and the existence of numerous frailty tools, some of which are challenging to use in the clinical setting (particularly those with objective measures) [12], investigating the association between fracture risk tools and frailty would be of interest. The fracture risk assessment (FRAX) tool, developed by the University of Sheffield and currently used internationally in the clinical setting, is online and computer-based [13]. It calculates 10-year fracture risk probabilities of hip and major osteoporotic fracture (MOF; inclusive of hip, clinical spine, forearm, proximal humerus) using routinely collected clinical risk factors in adults aged 40–90 years [14, 15]. FRAX was developed using primary data from nine population-based cohorts in multiple countries including centres in North America, Europe, Asia and Australia. Clinical risk factors for fracture that provide independent information on fracture risk were identified using a series of meta-analyses [14]. These clinical risk factors include age, current weight, height, prior low trauma fracture, parental hip fracture, current smoking, glucocorticoid use, rheumatoid arthritis, secondary osteoporosis and alcohol intake. These clinical risk factors are entered into the online tool and the algorithm generates 10-year fracture risk probabilities from the inputted data. Fracture probability may be calculated with or without BMD and, thus, an individual can have up to four probability scores: hip fracture 10-year probability scores with and without BMD, as well as MOF 10-year probability scores with and without BMD [13, 15]. The higher the FRAX score, the higher the fracture risk [15]. The FRAX algorithm has been validated in 11 independent cohorts and models have been calibrated for different countries using country-specific fracture and mortality rates [13, 14]. Currently there are FRAX models available for 31 countries [13].

Most studies have shown that frailty status predicts fracture risk, likely due to frailty and bone fragility having overlapping risk factors. Thus, the primary aim of the study was to investigate the association between the FRAX tool and frailty in cross-sectional analyses. The secondary aim was to explore the association between FRAX calculated at one time point and frailty measured 15 years later.

## Methods

### Participants

The current study utilised data from the Geelong Osteoporosis Study (GOS), a longitudinal population-based cohort study of randomly selected adults from the Barwon Statistical Division in south-eastern Australia. At baseline, 1494 women and 1540 men were recruited; a comprehensive description of the study has been provided elsewhere [16]. Data from two visits, 15 years apart, were used in this study. For the women, this was baseline (1993–1997) and the follow-up (defined as the 15-year visit) (2011–2014). For the men, this was also baseline (2001–2006) and follow-up (2016–2019). This study included two analyses. A cross-sectional, exploratory analysis was conducted, investigating the association between FRAX-scores and frailty at the 15-year follow-up visits for both women and men. Subsequently, secondary analyses were completed to investigate the association between FRAX-scores calculated at baseline and frailty over a 15-year period. Further details are described below.

Participants underwent assessment using dual energy x-ray absorptiometry (DXA), anthropometry and test of functional mobility in conjunction with questionnaire data concerning health and lifestyle behaviours [16]. The questionnaires and protocols for clinical measurements used to obtain these data were consistent for women and men at all visits.

### Cross-sectional analysis

**Fig. 1:**
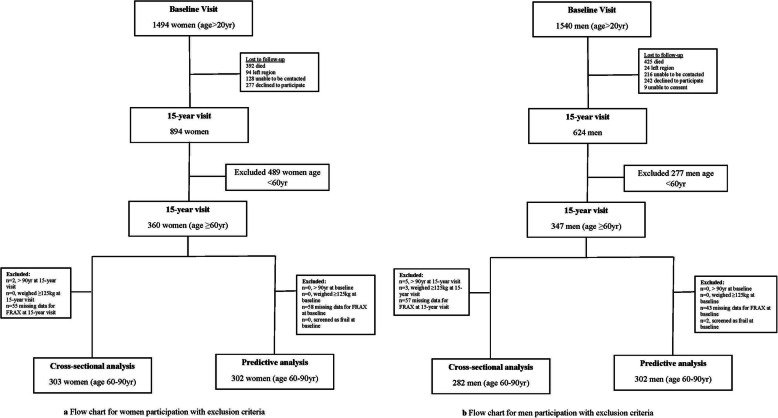
Flow charts for male and female participation and reasons for exclusion

In these analyses, 282 men and 303 women aged 60–90 years at the 15-year follow-up visit were included. The sampling process is shown in Figs. 1a and b.

### Secondary analysis

From the baseline visit for the women, 849 women (aged ≥20 years) returned for the follow-up, of whom 302 women (aged 41–75 years) were included in these analyses. The reasons for loss to follow-up included death (*n* = 392), migration from the region (*n* = 94), unable to be contacted (*n* = 128). Another 277 women declined to participate citing following reasons: personal reasons (e.g invasion of privacy, not interested (*n* = 124), old age/unable to cope (*n* = 78), time constraints (*n* = 36), illness (*n* = 21), too far to travel (*n* = 5), failure to keep appointment (*n* = 4), language barrier (*n* = 1) and for reasons not provided (*n* = 8) [17].

For the men, of the 624 men (aged ≥20 years) that returned for the 15-yr visit, 302 (aged 46–82 years) were included in these analyses. From baseline, 242 were lost to follow-up at the 15-year visit for the following reasons: personal reasons (*n* = 108), old age/unable to cope (*n* = 48), time constraints (*n* = 45), illness (*n* = 33), too far to travel (*n* = 2), failure to keep appointment (*n* = 4), language barrier (*n* = 1) and for reasons not provided (*n* = 8). Reasons for non-participation also included death (*n* = 425), migration from region (*n* = 24), unable to consent (*n* = 9) and unable to be contacted (*n* = 216) (Fig. 1a&b).

### Ethical considerations

All procedures performed in studies involving human participants were in accordance with the ethical standards of the institutional and national research committees and with the 1964 Helsinki declaration and its later amendments or comparable ethical standards. The study was approved by the Barwon Health Human Research Ethics Committee.

Written, informed consent was obtained from all participants in the study.

### Frailty assessment

At the 15-year follow-up, frailty was identified using a modified Fried frailty phenotype, which categorised participants into frail, pre-frail or robust groups [2]. This tool used five items; unintentional weight loss, exhaustion, low physical activity, weakness and slowness. Unintentional weight loss, exhaustion and low physical activity were ascertained by self-reported questionnaires. Weakness was determined using handgrip strength (HGS), measured using a hand-held Jamar device (Sammons Preston, Bolingbrook, IL, UK) for women and Vernier (Venier Software and Technology, Beaverton, USA) for men. To align our measurements as close as possible to the Fried criteria, HGS values for the men were transformed to Jamar equivalent values. This was done because the Fried criteria for frailty utilises cut-off points for muscle weakness from the Jamar device. The equation that was used was: *MeanHGS*_*Jamar*_(kg) = 8.68 + 0.840*MeanHGS_*Vernier*_(kg) + 8.31*Sex (where male = 1). The conversion equation was developed by measuring the maximum HGS on each device for 45 men and women aged 21–67 years. Weakness was defined by cut-points from the Fried phenotype derived from the lowest 20% stratified by sex and body mass index [2]. Slowness was measured using timed up and go (TUG) test. A score of greater than 10 s is considered as being slow [18]. Having three or more of these items categorised a person as frail, 1–2 as pre-frail, and none as robust.

For the secondary analyses, we sought to exclude participants who may have had any indication of frailty at baseline. As there were insufficient data to calculate the Fried phenotype at baseline, we used a modified Frailty Index of deficit accumulation that was constructed using guidelines from Searle et al. [16] but included only 18 health variables (Additional file 1 Table A.1). Those who had indications for frailty at baseline (*n* = 2 men only) were excluded from these analyses. Self-reported data were used in the construction of the index, with the exception of BMI (height and weight were measured, then BMI calculated) and high blood pressure, which was measured using a digital sphygmomanometer. High blood pressure referred to systolic blood pressure ≥ 140 mmHg and/or diastolic blood pressure ≥ 90 mmHg.

### Fracture risk assessment

FRAX (Australia) [15] was calculated using the following information; age, current weight, height, participants self-reported parental hip fractures, current smoking status, glucocorticoid use, rheumatoid arthritis. Secondary osteoporosis comprised type I (insulin dependent) diabetes, osteogenesis imperfecta in adults, untreated long-standing hyperthyroidism, hypogonadism or premature menopause (< 45 years), chronic malnutrition, or malabsorption and chronic liver disease. This information was captured by questionnaire, except for malnutrition where BMI < 18.5 kg/m^2^ was used as a proxy. Previous low trauma fractures were identified from radiological reports [16]. BMD at the femoral neck was measured using dual energy x-ray absorptiometry (DXA; Lunar Prodigy-Pro, Madison, WI, USA). Alcohol consumption was derived from the Victorian Cancer Council Food Frequency Questionnaire [19] and converted into a binary value (cut-point was alcohol consumption of ≥30 g/ day) for use in FRAX for all data except men 15-year follow-up, where it was identified by questionnaire [16]. Ten-year FRAX probabilities for MOFs and hip fracture with and without BMD were calculated using the online tool for each participant. Each participant had four scores generated, MOF FRAX scores without BMD (MOF-FRAX_noBMD_)_,_ hip FRAX score without BMD (hip-FRAX_noBMD_), MOF FRAX scores with BMD (MOF-FRAX_BMD_), and hip FRAX with BMD (hip-FRAX_BMD_). Only participants with complete data at either visit were included.

### Statistical analyses

Cross-sectional data analysis for examining differences in FRAX scores between the three frailty groups at the 15-year follow-up was completed using Kruskal-Wallis test for non-parametric data.

The secondary analyses investigated the association of FRAX at baseline and frailty at the 15-year follow-up. Area under Receiver operating characteristic (AUROC) curves and diagnostic characteristics tests including sensitivity, specificity and likelihood ratios were performed. This was done by examining the sensitivity and specificity for decile cut-points and selecting the cut-point with an optimal combination of sensitivity and specificity. Minitab (Version 18, State College, PA, USA) and STATA (Version 16, College Station, Texas, USA) statistical software were used for statistical analyses.

## Results

### Cross-sectional analysis

Descriptive characteristics are summarised in Table 1.

**Table 1:** Descriptive characteristics of men and women at the 15-year follow-up visit and stratified by frailty groups. Values reported as means ±SD or median (IQR)

	All	Frail	Pre-frail	Robust	***P***-value
**Women**	***N*** **= 303**	***N*** **= 47**	***N*** **= 168**	***N*** **= 88**	
Age (yr)	70 (65–75)	76 (70–84)	70 (65–75)	67 (63–72)	< 0.001
Weight (kg)	73.7 ± 15.0	73.0 ± 15.0	74.7 ± 15.0	72.4 ± 15.0	0.474
Height (m)	1.60 ± 0.06	1.57 ± 0.06	1.60 ± 0.06	1.61 ± 0.06	0.004
Body mass index (kg/m^2^)	29.0 ± 5.7	29.4 ± 5.5	29.4 ± 5.9	28.0 ± 5.3	0.141
Femoral neck bone mineral density (g/cm^2^)	0.835 ± 0.130	0.801 ± 0.136	0.844 ± 0.136	0.835 ± 0.108	0.127
FRAX scores^a^					
MOF-FRAX_BMD_		12.0 (5.7–17)	6.1 (3.2–10)	4.6 (3.0–8.1)	< 0.001
MOF-FRAX_noBMD_		14.0 (6.5–28)	7.5 (4.0–14.8)	5.9 (3.6–9.0)	< 0.001
Hip-FRAX_BMD_		3.8 (1.3–6.1)	1.45 (0.4–3.6)	1.0 (0.4–2.6)	< 0.001
Hip-FRAX_noBMD_		5.8 (2.0–13)	2.6 (1.0–6.6)	1.8 (0.9–3.6)	< 0.001
**Men**	***N*** **= 282**	***N*** **= 41**	***N*** **= 147**	***N*** **= 94**	
Age (yr)	71 (66–78)	78 (73–74)	71 (66–79)	68 (64–71)	< 0.001
Weight (kg)	82.7 ± 12.4	88.1 ± 13.2	82.0 ± 12.7	81.4 ± 11.09	0.011
Height (m)	1.72 ± 0.08	1.71 ± 0.08	1.72 ± 0.06	1.73 ± 0.10	0.510
Body mass index (kg/m^2^)	28.0 ± 4.3	30.1 ± 4.1	27.5 ± 3.6	27.5 ± 5.1	0.002
Femoral neck Bone mineral density (g/cm^2^)	0.935 ± 0.137	0.961 ± 0.153	0.921 ± 0.123	0.947 ± 0.149	0.158
FRAX scores					
MOF-FRAX_BMD_		4.2 (3.0–5.5)	3.5 (2.3–1.2)	2.7 (1.7–4.3)	< 0.001
MOF-FRAX_noBMD_		6.2 (4.0–7.3)	4.0 (2.5–7.1)	3.0 (1.1–4.7)	< 0.001
Hip-FRAX_BMD_		1.6 (0.8–2.3)	1.0 (0.5–2.2)	0.7 (0.3–1.6)	< 0.001
Hip-FRAX_noBMD_		3.1 (1.6–3.8)	1.6 (0.7–3.7)	0.9 (0.4–2.0)	< 0.001

^a^Fracture risk assessment (FRAX), Major Osteoporotic Fractures (MOF) FRAX score with Bone mineral density (BMD)(MOF-FRAX_BMD_), MOF-FRAX score without BMD (MOF-FRAX_noBMD_), hip FRAX with BMD (hip-FRAX_BMD_),)_,_ hip FRAX score without BMD (hip-FRAX_noBMD_)

### Women

Of 303 women, 47 (15.5%) were frail, 168 (55.4%) pre-frail and 88 (29.0%) robust at the 15-year follow-up. There were differences in age and height between the frailty groups, where median age increased across the frailty groups and the mean height decreased across the frailty groups. No differences in weight, BMI and BMD were detected between the three groups. The frail group had higher median scores for all four FRAX scores compared to the pre-frail and robust groups (Table 1).

### Men

Of 282 men, 41 (14.5%) were frail, 147 (52.1%) pre-frail and 94 (33.3%) robust at the 15-year follow-up. Frail men were older in age, weighed more and had a higher BMI compared to the pre-frail and robust groups; otherwise the groups were similar. Analysis revealed that the frail group had higher median scores for all four FRAX scores compared to the pre-frail and robust groups (Table 1).

### Secondary analyses

#### Women

In a non-response analysis, individuals at baseline who did not participate at the 15-year follow-up were found to be older, shorter and weigh less compared to those who participated (data not shown). At baseline, only one woman was identified with 25% deficits on the 18 health variables but none had greater than 25% of total deficits. Thus, no participants had indications of frailty at baseline (Additional file 1 Table A.1). FRAX risk factors for women at baseline, according to frailty status at 15-year follow-up, are summarised in Table 2. Prior fracture and glucocorticoid use were associated with frailty, with the frail group having higher proportions compared to the other two groups. There was also a correlation between frailty and age, height and BMD, where women in the frail group were older, shorter and had a lower BMD. The median time of follow-up between baseline and 15-year follow-up was 16.5 years (IQR 15.78–16.96). FRAX scores increased over time (data not shown).

**Table 2:** FRAX variables for men and women at baseline and according to frailty status at 15-year follow-up. Values reported as means ±SD, median (IQR), or n (%)

	ALL	Frail	Pre-frail	Robust	***p***-value
**Women**	***N*** **= 302**	***N*** **= 46**	***N*** **= 168**	***N*** **= 88**	
Age (yr)	53 (48–73)	62 (53–67)	53 (47–60)	51 (47–56)	**≤** 0.001
Weight (kg)	70.8 ± 13.1	71.7 ± 13.3	71.2 ± 13.2	69.6 ± 12.9	0.583
Height (cm)	161.3 ± 6.0	159.3 ± 5.2	161.3 ± 6.0	162.4 ± 6.1	0.018
Prior Fracture	31 (10.3)	11 (24.0)	13 (7.7)	7 (8.0)	0.004
Parental hip fracture	18 (6.0)	4 (8.7)	10 (6.0)	4 (4.5)	0.629
Smoking	33 (10.9)	3 (6.5)	22 (13.1)	8 (9.1)	0.362
Glucocorticoids	2 (0.6)	1 (2.2)	1 (0.6)	0 (0)	^**a**^
Rheumatoid Arthritis	24 (7.9)	8 (17.4)	14 (8.3)	2 (2.3)	0.009
Secondary Osteoporosis	36 (11.9)	4 (8.7)	25 (14.9)	7 (8.0)	0.204
Alcohol	2 (0.7)	0 (0)	0 (0)	2 (2.3)	^**a**^
BMD^b^ (g/cm^2^)	0.928 ± 0.146	0.876 ± 0.150	0.933 ± 0.153	0.946 ± 0.124	0.025
**Men**	***N*** **= 302**	***N*** **= 46**	***N*** **= 158**	***N*** **= 98**	
Age (years)	57 (52–65)	68 (60–76)	57 (66–52)	54 (51–59)	**≤** 0.001
Weight (kg)	83.7 ± 12.7	87.3 ± 12.9	83.0 ± 13.0	83.3 ± 11.9	0.108
Height (cm)	174.5 ± 6.4	173.7 ± 7.2	174.4 ± 6.1	175.0 ± 6.4	0.496
Prior Fracture	49 (16.2)	6 (13.0)	29 (18.4)	14 (14.3)	0.565
Parental hip fracture	23 (7.6)	3 (6.5)	15 (9.5)	5 (5.1)	0.417
Smoking	31 (10.3)	3 (6.5)	17 (5.6)	11 (11.2)	0.657
Glucocorticoids	1 (0.3)	0 (0)	1 (0.6)	0 (0)	^**a**^
Rheumatoid Arthritis	7 (2.3)	1 (2.2)	6 (3.8)	0 (0)	0.145
Secondary Osteoporosis	4 (1.3)	0 (0)	4 (14.9)	0 (0)	^**a**^
Alcohol	82 (27.2)	12 (26.1)	44 (27.8)	26 (26.5)	0.959
BMD (g/cm^2^)	0.982 ± 0.127	1.016 ± 0.150	0.962 ± 0.116	1.000 ± 0.127	0.009

^**a**^Too few to conduct statistical analysis

^b^ Bone mineral density (BMD)

In discriminating frailty groups, the AUROC analysis of FRAX scores demonstrated the greatest value for MOF-FRAX_BMD_ (0.72). Slightly lower values were observed for MOF-FRAX_noBMD_ (0.71). The FRAX scores for the hip displayed a similar pattern; hip-FRAX_BMD_ demonstrated a slightly increased AUROC curve compared to hip-FRAX_noBMD_ (0.69 and 0.68). The lowest AUROC values were observed for BMD alone (0.62). When comparing the association between FRAX tool and BMD alone with frailty, the association between FRAX tool was better as indicated by the AUROC curve (0.72 versus 0.62 respectively) (Table 3). Using AUROC analysis to determine preliminary cut-points for optimum sensitivity and specificity in determining frailty, the fifth decile was the optimal point for all FRAX scores, with MOF-FRAX_BMD_ having the optimal values based on AUROC curve (AUROC = 0.72, sensitivity = 80.0%, specificity = 45.7%,) (Table 3).

**Table 3:** Determination of Fracture Risk Assessment (FRAX) score cut-off points for screening for frailty using Area under the Receiver Operating Characteristic (AUROC) curves using sensitivity and specificity measures

FRAX scores	Area under the ROC curve (95% CI)	Cut point (Decile)	% Cut point for fracture risk ^a^	Sensitivity	Specificity	Predictive Value (%)	Positive Likelihood ratio (LR+)	Negative Likelihood ratio (LR-)
Women								
MOF^b^with BMD^c^	0.72 (0.63–0.80)	5	1.50 (1.06,1.94)	80.0%	45.7%	50.8	1.47	0.44
MOF without BMD	0.71 (0.62–0.79)	5	1.60 (1.21,1.99)	82.2%	45.0%	50.5	1.50	0.40
Hip with BMD	0.69 (0.60–0.78)	5	0.10 (0.00,0.38)	84.4%	30.2%	38.3	1.21	0.51
Hip without BMD	0.68 (0.60–0.77)	5	0.20 (0.02,0.38)	75.6%	49.2%	53.1	1.49	0.50
Men								
Hip without BMD	0.76 (0.68–0.82)	5	0.30 (0.12,1.94)	89.6%	45.7%	52.6	1.64	0.23
MOF without BMD	0.73 (0.66–0.80)	5	1.65 (1.36,1.94)	87.5%	47.7%	54.0	1.67	0.26
MOF with BMD	0.68 (0.60–0.76)	5	1.70 (1.45,1.95)	79.2%	46.5%	51.6	1.48	0.45
Hip with BMD	0.67 (0.59–0.75)	5	0.20 (0.05,0.35)	81.3%	44.1%	50.0	1.45	0.42

^a^Reported as median (95% CI) fracture risk percentage

^b^*MOF* Major Osteoporotic fractures

^c^*BMD* Bone mineral density

#### Men

Similar to the women, in the non-response analysis, participants that did not attend the 15-year follow-up visit were older, shorter and weighed less (data not shown). Among the men screened at baseline, only two showed indications of frailty with > 25% deficits of the 18 health variables (Additional file 1 Table A.1), and were excluded from the longitudinal data analyses. FRAX risk factors for men at baseline, according to frailty status at 15-year follow-up are summarised in Table 2. Age and BMD were correlated with frailty. The median time of follow-up between baseline and 15-year follow-up was 13.9 years (IQR 13.4–14.5). FRAX scores increased over time (data not shown).

The ROC analyses demonstrated that the area under the ROC curve was greater for FRAX scores than for BMD alone (Table 3). In descending order, hip-FRAX_noBMD_ had the greatest AUROC value (0.76), followed by MOF-FRAX_noBMD_ (0.73), then MOF-FRAX_BMD_ (0.68), then hip-FRAX_BMD_ (0.67) and BMD alone (0.57). Using the AUROC analysis to determine preliminary cut-points for optimum sensitivity and specificity in determining an association with frailty, the fifth decile was the optimal point for all FRAX scores, with hip-FRAX_noBMD_ having the optimal values based on AUROC curve (sensitivity = 89.6%, specificity = 45.7%) (Table 3).

## Discussion

This study demonstrates that men and women with frailty had the highest FRAX scores compared to the pre-frail and robust groups, indicating that FRAX is associated with frailty. Compared to using BMD alone, the FRAX tool showed a better association. For women, the difference in AUROC curves between MOF-FRAX scores with and without BMD was small. For men, FRAX scores without BMD were better overall, with hip-FRAX having a slightly higher area under the curve compared to MOF-FRAX score.

This study showed that frail women tended to have lower femoral neck BMD. These results are similar to previous studies that demonstrated that frail individuals had lower femoral neck BMD [20, 21]. In a study of community-dwelling older women, those who were frail at baseline using the Vulnerable Elders Survey (VES-13) had lower spine and hip BMD at 1 year follow up [22] whereas frail men tended to have higher BMD. These results are similar to those of Cook et al. [23] who reported femoral neck BMD was not lower in frail men; however, it should be noted that these data were derived using the Rockwood frailty index and not the Fried frailty phenotype. Our data suggest that FRAX scores are better associated with frailty compared to BMD alone. BMD was shown to be minimally associated with frailty. This may be explained by the FRAX tool’s inclusion of risk factors that are also associated with frailty [24]. These risk factors include age, weight and height, fractures, diabetes, malnutrition, smoking and alcohol [2, 25–29]. We hypothesise that malnutrition could lead to muscle loss, which could result in muscle weakness and fatigue as specifically outlined in the definition of frailty by Fried et al. [2].

In the secondary analyses, we identified most participants who went on to become frail (sensitivity 80%). However, our results also indicated a high number of false positives. Thus, the association between FRAX and frailty could be beneficial in enhancing the knowledge currently available for frailty.

Results of the current study revealed an association between FRAX and frailty. This information can enhance our understanding of frailty and open an exploratory avenue for research focusing on the utility of this tool. If this utility is possible, it would be advantageous, as it will utilise information that is readily available and allow screening and possible identification of those that are at risk of being frail, who can then be investigated further using validated tools. This might also permit targeting of interventions for delay or prevention of frailty development. While this tool is unidimensional in its functionality, addressing only the physical components of frailty, it has the advantage of being an online calculation tool, using readily available information [13].

Our study has a number of strengths including the random selection of participants from the general population, and the use of many objective measures in calculations of frailty scores. However, it should be noted that our sample is a nested cohort from a bigger study. Limitations of this research include the use of some self-reported information, loss to follow-up between the two time points and the use of a modified Fried phenotype to identify frailty. However, the prevalence of frailty using the modified Fried phenotype tool mirrors that of previous studies undertaken in populations with similar characteristics [2, 30, 31]. Data were collected at different time points for the men and women (5 years apart), by different research personnel; however, all personnel were trained and the same questionnaires and protocols for measurements were utilised at each visit. The age range for our study participants was limited to older adults, specifically between the ages of 40–90 years (due to FRAX age limitation); however, it should be noted that most studies on frailty use participants aged 60 years and older as this is the age range at higher risk of frailty [2, 12, 32]. We acknowledge that the older old participants (≥80 yr) are more likely to have more characteristics for frailty [33]. Between the two time points used, there was attrition due to death, relocation, non-respondents and withdrawal from the study. Our participants were generally healthy as they were from the general population. As such, we did not expect to have many frail individuals. If recruitment had been from an aged-care setting, then a larger number of frail individuals would likely have been identified. We recognise the potential for differential loss to follow-up on the basis of disability and the likelihood of a healthy survivor effect. While only two men had indications of frailty at baseline, we acknowledge the limitation of using the modified frailty index of deficit accumulation, as it only had 18 items rather than the recommended minimum of 30. The frailty index of deficit accumulation was used only to identify people for whom there was an indication of frailty because insufficient data were available at baseline to determine the Fried phenotype for the purposes of exclusion. However, within these constraints, an association was observed and we suggest further research into the utility of FRAX in the evaluation of frailty in larger cohorts. Finally, the current study investigated FRAX and frailty in a population-based sample of adults who are predominantly Caucasian (98%) and so further research is necessary to determine whether FRAX is associated with frailty in other culturally diverse populations.

## Conclusion

The results of this study show an association between FRAX and frailty cross-sectionally and over 15 years. Preliminary results suggest that FRAX could enhance the knowledge surrounding frailty. Further research using larger and more culturally diverse datasets will be required to determine the utility of this tool.

## Supplementary information

**Additional file 1.** Table A.1

## Data Availability

The datasets used and/or analysed during the current study are available from the corresponding author on reasonable request.

## Abbreviations

BMD: Bone mineral density
FRAX: Fracture risk assessment
MOF: Major osteoporotic fracture
GOS: Geelong Osteoporosis Study
DXA: Dual energy x-ray absorptiometry
HGS: Hand grip strength
TUG: Timed up and go
BMI: Body mass index
MOF-FRAX_noBMD_: Major osteoporotic fracture risk assessment scores without bone mineral density
hip-FRAX_noBMD_: Hip fracture risk assessment scores without bone mineral density
MOF-FRAX_BMD_: Major osteoporotic fracture risk assessment scores with bone mineral density
hip-FRAX_BMD_: Hip fracture risk assessment scores with bone mineral density
AUROC: Area under Receiver operating characteristic
IQR: Interquartile range
SD: Standard deviation
