# Supplementary material for: The association between a fracture risk tool and frailty: Geelong Osteoporosis Study
Source: BMC Geriatr. 2020 Jun 5;20:196. doi: 10.1186/s12877-020-01595-8 (PMC7275607; doi:10.1186/s12877-020-01595-8)
Supplement: Supplementary file 1 — Additional file 1. Table A.1 [file 12877_2020_1595_MOESM1_ESM.docx]

**Supplementary Data**

**Appendix Table A.1**

Variables used to screen for frailty at the baseline visit for men and women. Variables chosen based on Searle et al (26). Data presented as number of women/men screened as positive for each deficit.

| **Frailty variables** | **Women (N= 302)** | **Men (N=302)** |
| --- | --- | --- |
| Help Bathing | 0 | 0 |
| Help Dressing | 0 | 0 |
| Help getting in/out of chair | 0 | 0 |
| Help walking around house | 0 | 0 |
| Help eating | 0 | 0 |
| Help grooming | 0 | 0 |
| Help using Toilet | 0 | 0 |
| Help up/down stairs | 0 | 0 |
| Help with housework* | 0 | 0 |
| Help with meal preparations | 0 | 0 |
| Hypertension | 105 | 175 |
| Heart attack | 30 | 16 |
| Stroke | 0 | 7 |
| Cancer | 13 | 41 |
| Diabetes | 11 | 15 |
| Arthritis | 58 | 39 |
| Chronic lung disease | 20 | 11 |
| BMI (kg/m^2^)   - <18.5 | 0 | 0 |
| - ≥30 | 86 | 69 |
| - 25-29 | 105 | 137 |

*Only light housework considered
